# Peptidome analysis of human milk from women delivering macrosomic fetuses reveals multiple means of protection for infants

**DOI:** 10.18632/oncotarget.11532

**Published:** 2016-08-23

**Authors:** Xianwei Cui, Yun Li, Lei Yang, Lianghui You, Xing Wang, Chunmei Shi, Chenbo Ji, Xirong Guo

**Affiliations:** ^1^ From Nanjing Maternal and Child Health Medical Institute, Nanjing Medical University Affiliated Nanjing Maternal and Child Health Hospital

**Keywords:** human milk, macrosomic fetuses, peptidomics, antibacterial activity, adipocyte proliferation

## Abstract

Breastfeeding is associated with a lower incidence of obesity, diabetes, and cardiovascular disease later in life. While macrosomic infants have a higher risk of developing obesity and other metabolic disorders. Breast milk may contain special nutrients to meet the different growth needs of different infants. Whether mothers make breast milk different to meet the requirement of macrosomic infants is still unknown. Here, we conducted a comparison between mothers delivering macrosomic and non-macrosomic infants in colostrum endogenous peptides. More than 400 peptides, originating from at least 34 protein precursors, were identified by Liquid Chromatography/Mass Spectrometry (LC/MS). Out of these, 29 peptides found to be significant differently expressed (|fold change| ≥ 3, *P* < 0.01). Blastp analysis revealed 41 peptides may have established biological activities, which exhibit immunomodulating, antibacterial action, antioxidation, opioid agonist and antihypertensive activity. Furthermore, we found that peptide located at β-Casein 24-38 AA has antimicrobial effect against *E. coli*, *Y. enterocolitica* and *S. aureus*. While, κ-Casein 89-109 AA-derived peptide plays as a regulator of preadipocyte proliferation. The profile of endogenous peptides from macrosomic term infants is different from non-macrosomic terms. This different peptide expression potentially has specific physiological function to benefit macrosomic infants. Finally, we believe that our research is a meaningfull finding which may add to the understanding of milk peptide physiological action.

## INTRODUCTION

Pregnancies with a macrosomic infants gradually comprise a subgroup of high-risk pregnancies. As a common pregnant complication, macrosomia seriously threatens the health of pregnant women [1, 2]. These abnormal weight gain affects both healthy of the fetus and the mother. It is always accompanied with prolonged labour and abnormal haemorrhage during delivery, leading to perineal laceration and even cesarean section (CS) [3]. Meanwhile, these fetuses frequently bring with shoulder dystocia and brachial plexus palsy, even that such as severe birth asphyxia and neonatal mortality [4]. Macrosomic fetuses are at risk for metabolic abnormalities complications like electrolyte disturbances, glycopenia and hyperbilirubinaemia during neonatal period [5]. Among the long-term, obesity and metabolic disorders, cardiovascular disease and childhood cancers have to be mentioned [6–8].

As the most suitable food for the newborn, milk is characterized by its good for nutrition, easier to digest, immune regulating properties and anti-infective function, which all provide nutrition and protection for the infant during early life [6, 9, 10]. Recent research finds that breastfeeding helps to prevent the incidence of obesity, diabetes as well as cardiovascular disease later in life [11–13]. Hence, human milk is especially important for fetal macrosomias who are more likely than other infants to be obese later in life. Nowdays, scientists belive that the composition of breast milk is various according to different growth needs of different infants, such as preterm human milk is more suitable for babies born too early. So we suppose mothers may produce different milk for macrosomic and non-macrosomic infants, also these infants respond differently to the breast milk they drink.

Despite being known and studied for years, the low molecular weight (LMW) portion of proteome have never before attracted enough attention. In fact, this protein/peptide fraction is key factor that regulates many genes, participates in various pathways of disease and now deserves more and more attention these days [14–15]. Recent researchs have found a large number of functional endogenous peptides in the milk thus making human milk a carrier of biochemical messages [16–20]. Over 300 naturally produced peptides were identified originating from the protein composition of breast milk and antimicrobial activity of the peptide mixture was firstly considered [16]. David et al. (2014) did a comparison on the entire endogenous peptides from human milk and the milk gastric juice of term infants to investigate their componet difference [19]. The recognition of specific roles performed by milk endogenous peptides raises questions about whether women delivering macrosomic fetuses bring more benefits to macrosomic infants? Elucidation of the significance of the naturally occurring peptides in the milk to development in the human newborn remains to be further studied.

Here, we carried out a quantitative study on human milk endogenous peptides from macrosomic and non-macrosomic terms. The broad aim of this research was to develop an effective ultrafiltration method to purify naturally occurring human milk peptides for Liquid Chromatography/Mass Spectrometry (LC/MS) analysis. A main objective was to quantitatively compare the naturally occurring peptide profile of human milks collected from mothers delivering macrosomic and non-macrosomic infants. Another goal was to identify known peptide hormones in human milk of these different expression peptides. We further revealed that peptide located at β-Casein 24-38 AA (NQELLLNPTHQIYPV, henceforth called Casein 24) shows antimicrobial activities against *E. coli, Y. enterocolitica* and *S. aureus*. Peptide located at κ-Casein 89-109 AA (PHAQIPQRQYLPNSHPPTVVR, henceforth called Casein 89) exhibits growth inhibiting activity in human preadipocytes.

## RESULT

### Maternal and neonatal characteristics

Table 1 shows the basic characteristics of maternity and infant involved in this test. All recruited women belong to the same age group and have similar body mass index (*P > 0.05*). The macrosomic infants' body weight exhibits significant difference when compared with control group (*P < 0.05*). Further more, both 1 h and 2 h oral glucose tolerance test (OGTT) levels were significantly higher in macrosomic infants group.

**Table 1:** Maternal and neonatal clinical characteristics

Clinical Features	Macrosomia (*n*= 6)	Control (*n* = 6)
Maternal age (mean ± SD)	30.8 ± 1.4	29.7 ± 1.8
Maternal BMI (mean ± SD)	31.3 ± 0.6	26.3 ± 1.9
Week gestation (mean ± SD)	36.2 ± 0.8	36.4 ± 0.5
Mode of devliviery CS (%)	100 %	100 %
Brith weight (g) (mean ±SD)	4120.5 ± 54.3^a^	3220.5 ± 26.9
OGTT fasting	5.7 ± 0.3	4.7 ± 0.2
1 h	9.5 ± 0.3^a^	5.3 ± 0.3
2 h	7.2 ± 0.4^a^	4.9 ± 0.5

a *P* < 0.05 when compared with control group.

BMI, body mass index.

SD, standard deviation.

### Sample preparation

Mucins, caseins, and whey proteins are the leading member of the human milk total protein contents, the peptides account for only a small fraction. Therefore, ultrafiltration using molecular weight cut-off (MWCO) filters was used to enrich the peptide part. Studies on plasma and cerebrospinal fluid reveal that peptides always adsorbed on proteins surface and may therefore reduce the efficiency of ultrafiltration separation [21, 22]. Denaturing and reducing solution (7 M urea, 2 M thiourea, and 20 mM DTT) was used in order to disrupt the protein–peptides interactions. Mass spectrum analysis confirmed that the method can identify more peptides than prior studies on endogenous peptides [16–18].

### Peptide identification and quantitative analysis

Peptides compostion of the pooled human milk from mothers delivering macrosomic and non-macrosomic infants were directly analyzed by LC-MS after isotopomeric dimethyl labeled. More than 400 peptides, originating from at least 34 protein precursors, were identified from the six individuals (Table S1). Hierarchical clustering analysis showed a remarkable peptide expression profile change between the two compared groups (Figure S1). Of these peptide, 15 peptides presented high levels (a fold change ≥ 3.0, *P < 0.05*) in the milk from mothers delivering macrosomic infants, while 14 peptides accounted for the main composition of peptides in the milk from mothers delivering non-macrosomic infants (a fold change ≤−3.0, *P < 0.05*) (Table 2).

**Table 2:** Part peptides that are differentially expressed in human milk from women delivering macrosomic infants

Sequence	Mass	Protein Names	log_2_(M/H)	*P*-value
**Peptides down-regulated in human milk from women delivering macrosomic infants (> 3 folds)**				
LGSAMQNTQNLLQMPY	1807.9	Alpha-2-macroglobulin	−5.40	9.82E-16
LWSVPQPKVLPIPQQV	1828.1	Beta-casein	−4.62	2.82E-02
LPIPQQVVPYPQRAVPVQ	2028.2	Beta-casein	−3.87	5.24E-05
VPQPIPQTLALPPQP	1594.9	Beta-casein	−3.68	1.24E-05
DLENLHLPL	1062.6	Beta-casein	−3.50	4.83E-17
LALPPQP	734.4	Beta-casein	−3.48	2.53E-05
PHAQIPQRQYLPNSHPPTVVR	1745.9	Kappa-casein	−3.44	4.92E-02
GRVMPVL	770.4	Beta-casein	−3.27	3.24E-05
WRKMCRKLLDMTFSSKTNTLVVR	2869.5	vWF-cleaving protease	−3.26	4.81E-11
MSRLEVVFTALMNSK	1724.9	Cholesteryl ester transfer protein	−3.19	3.24E-05
DTVYTKGRVMPVL	1477.8	Beta-casein	−3.18	5.40E-05
LLLNPTHQIYPVTQPLAPVH	2250.3	Beta-casein	−3.18	5.30E-04
LLLNPTHQIYPVTQPLAPVHNPISV	2760.5	Beta-casein	−3.06	2.62E-02
VLPIPQQVVPYPQRAVPVQALL	2424.4	Beta-casein	−3.03	1.53E-05
**Peptides up-regulated in human milk from women delivering macrosomic infants (> 3 folds)**				
LALPPQPLWSVPQPK	1670.0	Beta-casein	3.11	1.54E-06
LQPLMQQVPQPIP	1487.8	Beta-casein	3.54	1.53E-05
NQELLLNPTHQIYPV	1063.6	Beta-casein	3.72	2.52E-02
VMPVLKSPTIPFFDPQIPKLTDLEN	2838.5	Beta-casein	3.93	4.83E-07
LWSVPQPKVLPIPQQVVPYP	2284.3	Beta-casein	4.06	5.44E-05
LLNPTHQIYPVT	1394.8	Beta-casein	4.17	2.54E-06
SPTIPFFDPQIPKLTDLEN	2171.1	Beta-casein	4.19	2.53E-05
ILPLAQPAVVLPVPQPEIMEVPKA	2548.5	Beta-casein	4.26	2.52E-02
PHAQIPQRQYLPNSHPPTVVR	1745.9	Kappa-casein	4.44	4.92E-02
SIQLPTTVRDIMNRW	1829.0	Membrane alanine aminopeptidase variant	4.54	5.27E-03
ASQLMGENRTMTIHNGMFFST	2372.1	Fibrinogen beta chain	5.09	4.92E-02
EDLIDEDDIPVRSFFP	1905.9	C4A variant protein	5.23	2.74E-03
ATSSLCSVTNTSMMTSE	1805.7	Ascites sialoglycoprotein	5.31	4.51E-03
LAQPAVVLPVPQPEIMEVPK	2154.2	Beta-casein	6.29	1.12E-09
MKFISTSLLLMLLVSSLS	1982.1	B cell-attracting chemokine 1	7.46	1.13E-12

M = Peptide Intensity (Human milk from women delivering macrosomic infants).

H = Peptide Intensity (Human milk from women delivering no-macrosomic infants).

### Characteristics of peptides identified from human milk

**Figure 1:**
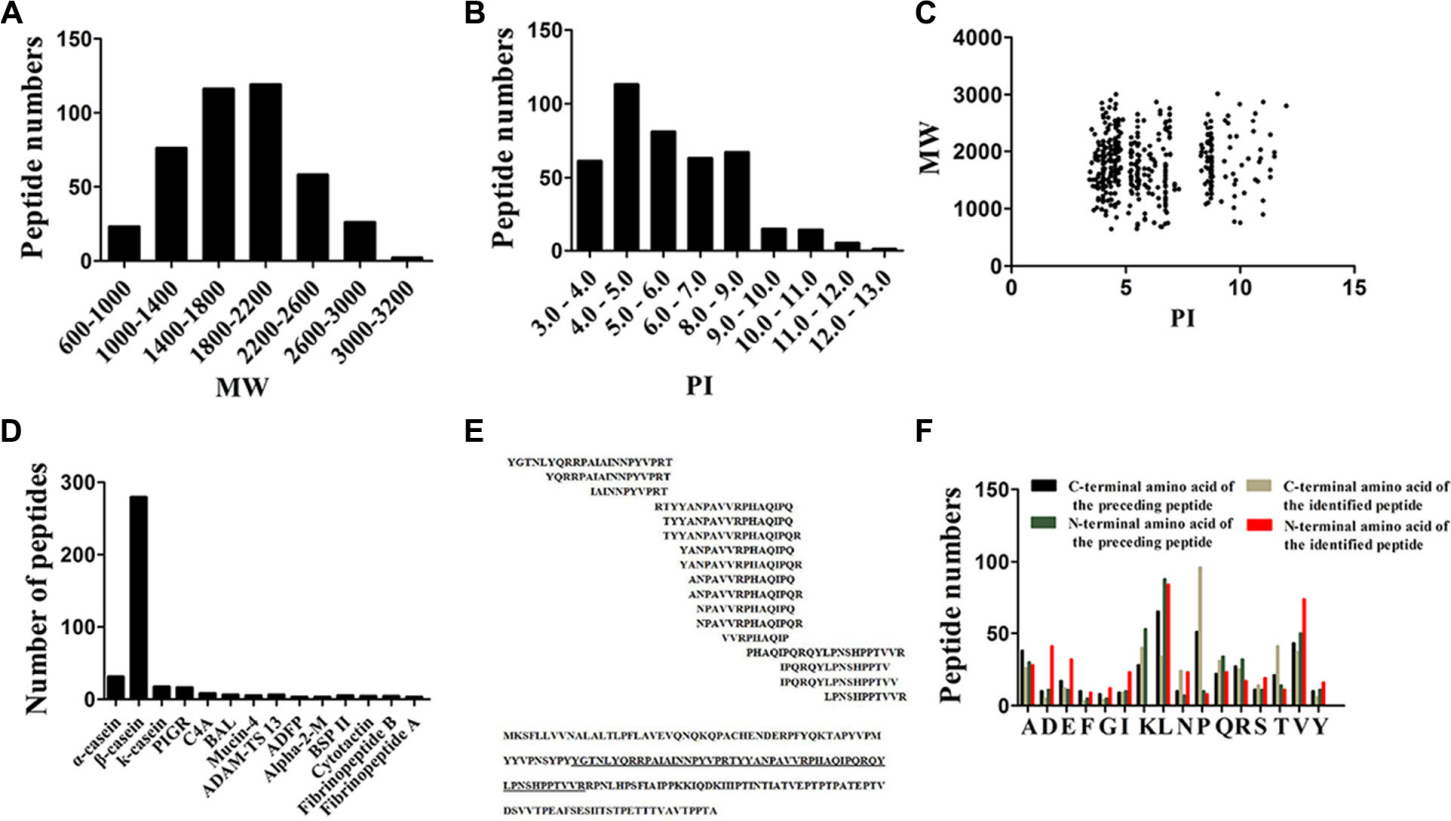
Characteristics of Peptides identified by Liquid Chromatography/Mass Spectrometry (LC/MS) (**A**) Distribution of molecular weight (MW) of peptides identified of colostrums samples milk. (**B**) Distribution of molecular pI of peptides identified of colostrums samples milk. (**C**) Scatter plot of MW versus isoelectric point (PI) distribution of the peptides. (**D**) Peptide numbers for each milk protein precursors. (**E**) Selected peptide ladder sequence and the locations of the 17 ladders identified in κ-casein. (**F**) Distribution of the four cleavage sites in the identified peptides.

Fristly, we analyzed the general features of the milk peptides identified from human milk. Both the molecular weight (MW) and isoelectric point (PI) of these peptides spread over a wide range, but we can observed that most of the peptides focused between 14–22 KDa and a acidic PI range (4.0–7.0), as shown in Figure 1A and 1B. Moreover, the relative distribution of PI versus MW also has a certain unique. Points of these identified peptides mainly gather into three groups, which distributed around PI 4, PI 6 and PI 10 (Figure 1C).

### Cleavage site patterns of identified endogenous peptides

A typical characteristic of these identified peptides was 279 peptides broken from protein of β-casein, occupied the highest proportion of the endogenous peptides from human milk (Figure 1D). Other peptides mostly derive from human milk proteins such as α-casein (CASA), κ-casein (CSN3), Polymeric immunoglobulin receptor (PIGR), Complement C4-A (C4A), Bile salt-activated lipase (BAL), Mucin-4 (MUC4), A disintegrin and metalloproteinase with thrombospondin motifs 13 (ADAM-TS 13), Perilipin-2 (ADFP), Alpha-2-macroglobulin (Alpha-2-M), Mothers against decapentaplegic homolog 1 (BSP II), Cytotactin (TNC), Fibrinopeptide B (FGB) and Fibrinopeptide A (FGA) (Figure 1D). In the further analysis, we observed that many of the peptides come from the same segment of one protein, which was in consistent with the observation in the serum peptidome analysis [23]. Another easily cleaved protein by the endogenous enzymes, κ-casein, generated 17 peptides in our case. However, the distribution of these peptides sequences were hardly a random, which arrayed like a ladder and came from 1 segments of κ-casein. The locations of the 17 peptide ladders identified in κ-casein were marked in Figure 1E.

We further combined peptidome analysis of human milk by LC/MS with bioinformatic analysis of the cleavage site distribution at the amino terminal end (N-terminal) and carboxyl terminal end (C-terminal) of the observed endogenous peptides. Briefly, all these four cutting sites of all the identified peptides were used to investigate the nature of proteolytic enzymes within human milk. Results showed that leucine (L) was the most popular C-terminal post-cleavage site of the preceding peptide, while proline (P) dominated the cleavage site of C terminus of the identified peptide. Leucine (L) is the most cleavaged site of N terminus of the identified peptide and N-terminal pre-cleavage site (Figure 1F).

### Putative functional peptides

Previous literatures showed the known human milk functional peptides have various biological effects such as immunomodulating (QPTIPFFDPQIPK) [24], antibacterial (QELLLNPTHQYPVTQPL/APVHNPISV) [25], antioxidant (WSVPQPK) [26], opioid agonist (YPFVEPI/YPFVE) [27], antihypertensive (HLPLP) [26]. Table 2 summarizes some peptides in human milk which had been report with potential beneficial effect on human health. We are surprised that 41 of the endogenous milk peptides shared > 50 % homology with known functional peptides, promising similar beneficial effects (Table 3). Of these peptides twelve matched known immunomodulatory sequences, eighteen matched antibacterial sequences, four matched antioxidant sequences, four matched opioid agonist sequences and three matched antihypertensive sequence (sequences shown in Table 3). It's worth noting that all these 41 peptides are primarily from β-casein. More research is needed for the remaining unknown function peptides.

**Table 3:** Putative functional peptides derived from human milk

Identified peptides	Precursor protein	Fragment	Functional peptides	Known activity
			QPTIPFFDPQIPK	Immunomodulating
FDPQIPKLTDLEN	Beta-casein	125−137		
FDPQIPKLTDLENL	Beta-casein	125−138		
GRVMPVLKSPTIPFFDPQIP	Beta-casein	111−130		
GRVMPVLKSPTIPFFDPQIPK	Beta-casein	111−131		
IPFFDPQIPKLTDLEN	Beta-casein	122−137		
MPVLKSPTIPFFDPQIP	Beta-casein	114−130		
MPVLKSPTIPFFDPQIPK	Beta-casein	114−131		
SPTIPFFDPQIPK	Beta-casein	119−131		
SPTIPFFDPQIPKLT	Beta-casein	119−133		
SPTIPFFDPQIPKLTDLEN	Beta-casein	119−137		
VLKSPTIPFFDPQIPK	Beta-casein	116−131		
VMPVLKSPTIPFFDPQIPK	Beta-casein	113−131		
			QELLLNPTHQYPVTQPL APVHNPISV	Antibacterial
ELLLNPTHQIYPVTQPLAPV	Beta-casein	41−60		
ELLLNPTHQIYPVTQPLAPVHNPIS	Beta-casein	41−65		
LLLNPTHQIYPVTQPLAPVHNPIS	Beta-casein	42−65		
LLLNPTHQIYPVTQPLAPVHNPISV	Beta-casein	42−66		
LLLNQELLLNPTHQIYPVTQPLAP	Beta-casein	36−59		
LLLNQELLLNPTHQIYPVTQPLAPV	Beta-casein	36−60		
LLNPTHQIYPVTQPLAPVHNPIS	Beta-casein	43−65		
LLNPTHQIYPVTQPLAPVHNPISV	Beta-casein	43−66		
LLNQELLLNPTHQIYP	Beta-casein	22−37		
LLNQELLLNPTHQIYPV	Beta-casein	22−38		
LNPTHQIYPVTQPLAPVHNPIS	Beta-casein	44−65		
NPTHQIYPVTQPLAPVHNPIS	Beta-casein	45−65		
NPTHQIYPVTQPLAPVHNPISV	Beta-casein	44−66		
NQELLLNPTHQIYPV	Beta-casein	24−38		
QIYPVTQPLAPVHNPISV	Beta-casein	49−66		
VTQPLAPVHNPISV	Beta-casein	53−66		
YPVTQPLAPVHNPIS	Beta-casein	51−65		
YPVTQPLAPVHNPISV	Beta-casein	51−66		
				
LWSVPQPK	Beta-casein	8−15	WSVPQPK	Antioxidant
LWSVPQPKVLPIP	Beta-casein	8−20		
LWSVPQPKVLPIPQQ	Beta-casein	8−22		
LWSVPQPKVLPIPQQV	Beta-casein	8−23		
			YPFVEPI/YPFVE	Opioid agonist
IYPFVEPIPYGFLPQN	Beta-casein	64−79		
QPQPLIYPFVEPIP	Beta-casein	59−72		
YPFVEPIPYGFL	Beta-casein	65−76		
YPFVEPIPYGFLP	Beta-casein	65−77		
			HLPLP	Antihypertensive
DLENLHLPL	Beta-casein	46−54		
DLENLHLPLP	Beta-casein	46−55		
DLENLHLPLPLL	Beta-casein	46−57		

### Antimicrobial activity of Casein 24

**Figure 2:**
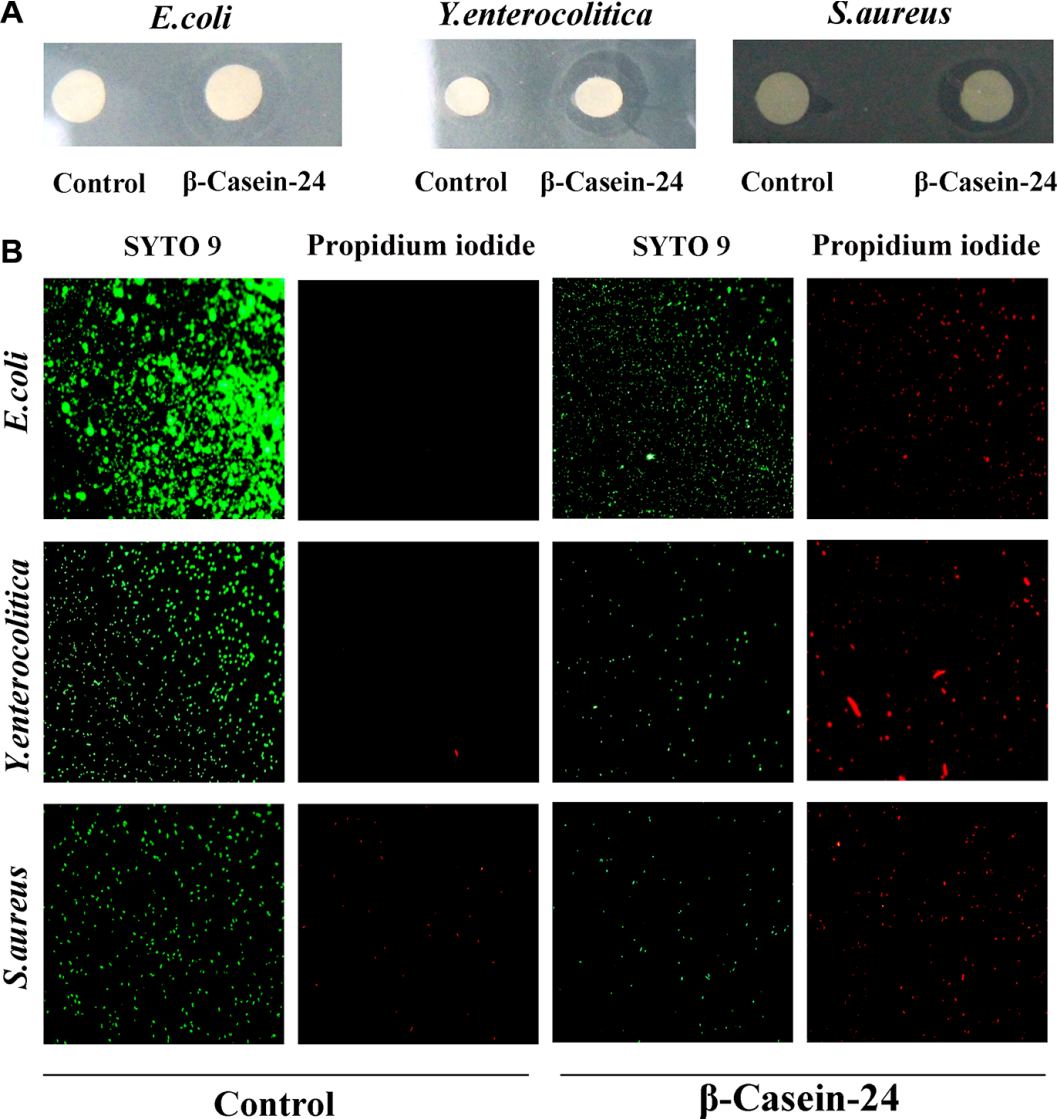
Casein 24 antimicrobial activity against *E. coli, Y. enterocolitica and S. aureus.* (**A**) Agar well diffusion assay. (**B**) Microscopic assessment of Casein 24 antimicrobial activity. Live cells emit green fluorescence and dead cells appear red (due to propidium iodide uptake).

Minimal inhibitory concentration (MIC), agar well diffusion and microscopic assessment experiments were employed for the investigation of Casein 24 antimicrobial activity against *E. coli, Y. enterocolitica* and *S. aureus*. Casein 24 exhibited antimicrobial activity against all the test strains (Figure 2A). The MIC values of Casein 41 against the bacterial strains are summarized in Table 4. The microscopic assessment of Casein 24 against the bacterial strains were showed in Figure 2B. Of the test stain, *E. coli* and *Y. enterocolitica* were more sensitive to Casein 24. Intensive research will be carried out in the future.

**Table 4:** The minimal inhibitory concentration of Casein 41 to selected microorganisms

	Microorganisms		
	*S. aureus*	*K. pneumoniae*	*E. coli*
**Minimum inhibitory concentration (μM)**	12.5−25	25	12.5−25

### Effect of Casein 89 on human preadipocyte proliferation

**Figure 3:**
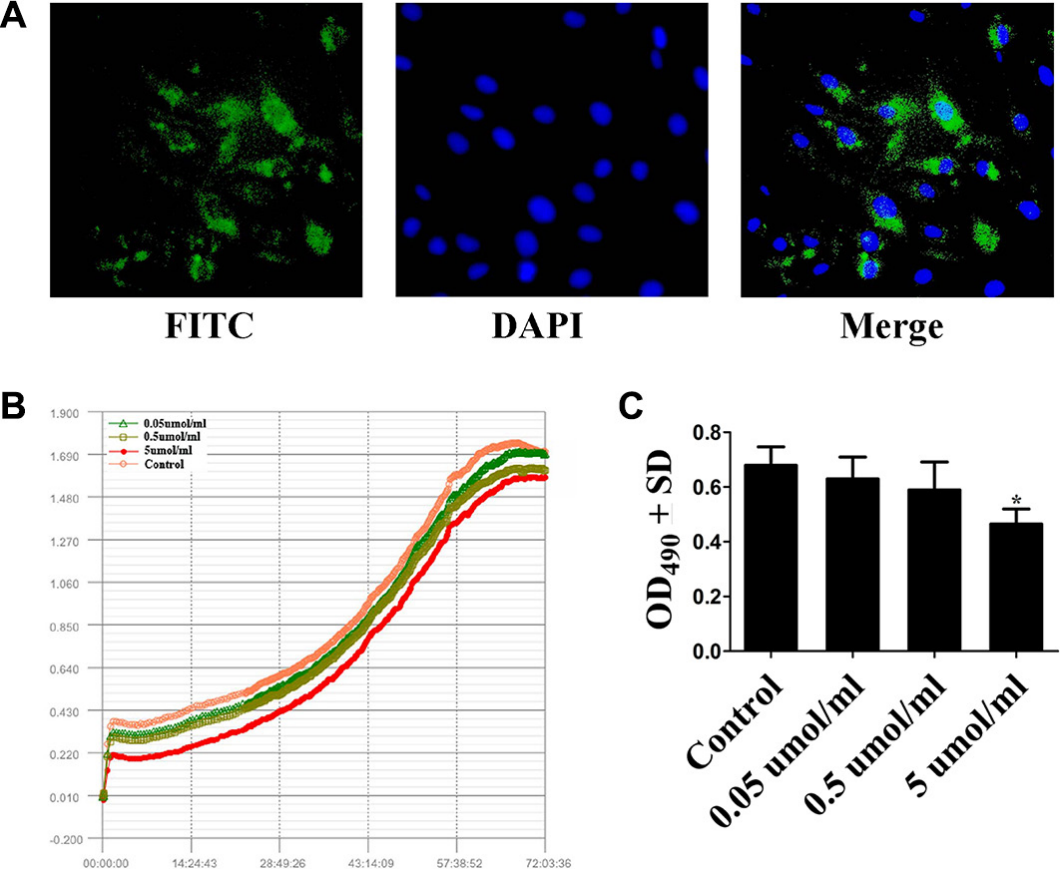
Cell Proliferation Assays of Casein 89 (**A**) FITC-labelled Casein 89 could successfully get into the preadipocytes. (**B**) Real-Time Monitoring of Casein 89 effect on human preadipocytes. (**C**) CCK-8 cell proliferation assay for Casein 89 treatment. Data was expressed as mean ± SD. *P < 0.05, when compared with control group.

Study on multifunctional peptides from human milk promots κ-casein derived peptides exhibit growth-promoting effect on 3T3-L1 cell (Mouse embryonic fibroblast cell line) [28]. Also, our peptide quantitative analysis revealed κ-casein derived peptide Casein 89 specially high-expressed in macrosomic group. To gain more experimental result supports, we investigated whether Casein 89 has a stimulating or inhibiting activity in human preadipocytes. We firstly provened that FITC-labelled Casein 89 could successfully get into the preadipocytes (Figure 3A). Its further effect was examined by incubating human preadipocytes with Casein 89 at different concentrations (0.05 μmol/ml, 0.5 μmol/ml and 5 μmol/ml), and measuring viability by the CCK-8 assay and xCELLigence^®^ impedance-based cell analysis system. CCK-8 assay and xCELLigence assays revealed that the absolute numbers of living cells were reduced in Casein 89 treated cultures compared to control cultures at 72 h (Figure 3B). We observed a dose dependent moderate inhibition to Casein 89 treatment (Figure 3C).

## DISCUSSION

A subfield of proteomics named peptidomics [29], which studies endogenous peptides that occur in body fluids, tissues, and cell lines, has recently increased. In present study, we conducted a comparison between mothers delivering macrosomic and non-macrosomic infants in colostrum endogenous peptides. To efficiently identify these naturally occurring peptides from human milk, a novel comprehensive approach was invented. Centrifugal ultrafiltration strategy is a well-established method based on a size-exclusion mechanism for concentrating and purifying peptide [30, 31]. Finally, more than 400 peptides were identified in both groups. Compared with previous studies on endogenous milk peptides [16, 18], more peptides were identified in this study (over 400 peptides vs 248 peptides, over 400 peptides vs over 300 peptides). This prompts ultrafiltration could be a more effective approach for endogenous peptides preparation.

Previous studies on endogenous peptides mainly focused on the proteolytic enzymes cleaving profile of the hydrolysed milk protein product [16, 18, 19], but lack a quantitative analysis of the complete protein-released peptidome, especially its correlation with disease progression. In fact, the quantitative description of peptides levels and their alterations under various physiological or pathological conditions, may reflect the biological processes occurring in body, are inevitably related to the proteolytic enzymes activities, and hus might be served as diagnostic biomarkers of pathological state. In this study, stable isotope dimethyl labeling was applied to quantitative the peptide composition of human milk from mothers delivering macrosomic and non-macrosomic infants [20], to uncover whether the human milk peptides amounts ranging from colostrum stage to mature milk stage. Results showed that 28 peptide were present at a statistically significant changes, accounting for about 7% of the total identified peptides.

Proteolytic activities of proteases in milk contribute to the production of smaller fragments such as peptides in milk [31]. Such peptides could become active only after releasing from their parent protein and this could attribute to gastric and pancreatic enzymes digestion process [32, 33]. The beneficial health effects of milk protein-derived bioactive peptides have been proved in many articles, which exhibit antimicrobial, antihypertensive, antioxidative, antithrombotic, mineral binding, immunomodulatory and opioid activities, among others [32, 34–36]. The function of these peptides is based on their amino acid composition and sequence signature. The high sequence homology between the naturally produced milk peptides with those reviewed peptides in previous articles suggests the likely function of these peptides. In this study, blastp analysis revealed high homology to query sequence with identities more than 50%. Of these peptides, twelve matched known immunomodulatory sequences, eighteen matched antibacterial sequences, four matched antioxidant sequences, four matched opioid agonist sequences and three matched antihypertensive sequence. The functional peptides may be specifically releasing to benefit macrosomic infants health and ensures safe growth and development during the early stages of their life.

Infection of newborns, as a major health care issue, is the leading cause of infants mortality during birth, while breast milk fed infants are at lower risk for infections [37, 38]. Early human milk feedings could provide protection for the newborn against these infection. However, the biological function which peptides widely dispay is its antibacterial effect. Combined with previous bioinformatic analysis, we are pretty sure milk endogenous peptides could play an important role in anti-infection. Experiments in our study confirmed that Casein 24 has a bacteria-fighting powers against *E. coli, Y. enterocolitica* and *S. aureus.* A previous study on pathogens inveterate that *E. coli, Y. enterocolitica* and *S. aureus* were the major strains in our neonatal intensive care unit (NICU) [39]. Nowdays, many gram negative pathogens show multidrug-resistant, including organisms that express extendedspectrum beta-lactamases such as *K. pneumoniae*, *K. oxytoca*, and other enterobacteriaceae such as *E. coli*. With the abusive application of a large number of antibiotics, antibiotics are subjected to certain constraints for bacterial resistance and screening bottleneck of new antibiotic. In this context, antimicrobial peptides promise to be the most ideal alternative medicine.

Also, current evidences indicate that the bacterial species of gut microbiota has been proposed to be a contributory factor in the development and maintenance of children and adolescent obesity [40, 41]. Abundant species of endogenous antimicrobial peptides were revealed in human milk of our study, especially the antimicrobial effect of Casein 24 was confirmed. So we speculate that these nature occurring peptides has a significant effect on gut microbiota composition, then influences host metabolism, adiposity and obesity complications. Perhaps, these effects of peptides could provide a protection for macrosomia.

Obesity and related metabolic diseases has become an epidemic [42]. In general, the offspring of obese women are more likely to be macrosomic than these offspring from their lean counterparts [43]. So inhibiting the proliferation of human preadipocyte may be a better regulation of the fetal macrosomia weight gain. During the last decade, peptides have been shown to exert a variety of metabolic effects. Natriuretic peptides could effective degrade lipids in adipose tissue [44], it has been proved that its plasma levels are negatively related to the development of insulin resistance and metabolic syndrome [45]. Several researches have revealed the potential therapeutic role of Angiotensin-(1–7) on treating and preventing metabolic disorders including both lipid and glucose disorders [46]. In consistent with the above studies, our studied showed that macrosomic group specially high-expressed peptide Casein 89 exhibit growth inhibiting activity in human preadipocytes.

It should be noted that this study also had some limitations. First, macrosomia occurs mainly in pregnancies complicated by Type-1 diabetes, Type-2 diabetes and gestational diabetes (GDM) [47, 48]. But during sample collection, the influence of diabetes and GDM dieases were excluded. Second, protection effects of milk endogenous peptides in macrosomia must be work on a whole, while integrated research on these peptides are lacking. Third, human milk will inevitably experience a process of digestion in gastrointestinal tract after taking, which peptides could escape digestion and present in blood stream are still unknown. Further more, the effects of endogenous peptides must be varied and we merely revealed its function from antimicrobial activity and inhibiting adipocyte hyperplasia sides.

In summary, we investigated the peptides profile in human milk from mothers delivering macrosomic infants, as well as detecting abundance changes of these peptides. Knowledge gained from these peptides in human milk will provide some insights into the regulatory mechanism involved in immunomodulating, antibacterial, antioxidant, opioid agonist, and developmental advantages on macrosomic infants from breastfeeding. It has been shown for the first time, that naturally occurring peptides in milk from macrosomic group, which show inhibiting properties on adipocytes. We believe that our research is a meaningfull finding which may add to the understanding of milk peptide physiological action for interested researchers.

## MATERIALS AND METHODS

### Sample collection

Colostrums from women delivering macrosomic infants (*n* = 6) and their matched controls (*n* = 6) were obtained at Nanjing Maternal and Child Health Hospital, China. Patients members were fully informed of the research and signed medical informed consent and this study was approved by Nanjing Medical University, Human Research Ethics Committee and Nanjing Maternal and Child Health Hospital. 10–20 ml of milk was collected using a mechanical breast pump (Ameda Egnell, Basel, Switzerland) by each lactating mother in the first 72 hours after birth. The milk was immediately stored on dry ice during transport to the laboratory (up to 1 h), and then centrifuged (1000 g, 20 min, 4°C) to remove the lipid layer and cell debris pellet. The aqueous phase (skim milk) was then added aliquot of protease inhibitor mixture (Complete Mini EDTA-free, Roche, Basel, Switzerland) and stored at −80°C.

### Sample treatment

Samples were then centrifuged at 120,00 g at 4°C for 30 min after thawing on the ice, and the supernatant was collected. Protein concentrations of all the samples were determined by the bicinchoninic acid (BCA) method (Pierce, Rochford, USA), using BSA as a standard. In the ultrafiltration method, 50 μL of milk samples were two-fold diluted in a denaturing and reducing solution (7 M urea, 2 M thiourea, and 20 mM DTT), and transferred to centrifugal filter devices. Molecular weight cut-off filters (Millipore, Billerica, MA, USA) of 10 kDa were washed with 0.5 ml H_2_O prior to use. The milk samples were centrifuged through the filters according to the manufacturer's recommendations. The filtrates were then desalted and concentrated by C18 solid phase extraction (SPE) (Strata C18-E, 55 μm, 760A, 100 mg/mL, Phenomenex, Torrance, CA, USA), and finally lyophilized. The derived peptides of the different samples are then labeled with isotopomeric dimethyl labels [20]. The labeled samples were mixed and simultaneously analyzed by LC-MS/MS whereby the mass difference of the dimethyl labels was used to compare the peptide abundance in the different samples.

### Liquid chromatography/mass spectrometry (LC/MS)

The freeze dried peptides was dissolved in 0.1% formic acid and filtered through a 0.45 μm membrane before injection. Reverse-phase chromatography was performed using a LC Packings C18 trap column (Acclaim PepMap100, 75 μm × 20 mm,) and separated with a LC packings C18 column (Acclaim PepMap, 75 μm × 150 mm) coupled to an Ultimate 3000 nano-LC system (Eksigent Technologies, Dublin, CA). The mobile phase was composed of: (A) 0.1% formic acid in water and (B) 0.1% formic acid in acetonitrile. A linear gradient from 0% to 20% B delivered at 200 μl/min over 50 min was used. The eluate was monitored by absorbance at 280 nm and five fractions were collected. The samples were directly injected into a MALDI TOF/TOF (Ultraflextreme, Bruker Daltonics, Bremen, Germany) instrument operated in the positive ion mode, following the previous strategy [49]. Full scan analysis was performed over the m/z range 400–5000 at 3 spectra/s. The capillary voltage and cone voltage were maintained at 3.9 kV and 40 V, respectively. For MS analysis, 2,000 single shot spectra were accumulated from 10 random positions on each sample, irradiating each position with 200 laser pulses.

The MS data were searched using the Mascot database search (http://www.matrixscience.com) against the SwissProt sequence database (http://www.expasy.org/tools/) considering the following variable modifications: phosphorylation, carbamylation, deamidation, methionine oxidation, acetylation, sulfation, and oxidized as well as reduced cysteines [15, 50]. The mass tolerance for precursor ion masses was set to 100 ppm and for miss cleavage, a single amino acid. All included peptides in the search were at least 6 amino acids long. PEAKS software (version 7.0, Bioinformatics Solutions) was also used to search the databases using MS/MS spectral data.

### Search for potential bioactive peptides

In order to further uncover the function of the specific peptides, we refered to the method as David et al. (2013) provided [16]. We first search for the known functional peptide in previous studies, then compared these identified peptides to each breast milk peptide using DNAman software. We also considered 50% indentity of the query sequence as a retaining minimum standard, so as to remove false positives.

### Antimicrobial activity assays of Casein 24

Bioinformatics analysis prompted Casein 41 has potential antimicrobial activity. The chemical synthetic peptide Casein 41 was purchased from Science Peptide Biological Technology CO.LTD (Shanghai, China). Then, the antimicrobial action of Casein 41 was tested against *S. aureus, K. pneumoniae* and *E. coli* using minimal inhibitory concentration, agar well diffusion assay and microscopic assessment experiment. Briefly, these three microorganisms were cultured overnight, so as to enhance the activity of bacteria. MIC was determined in 96-well polypropylene microtitre plates by concentration-dependent reducing Casein 41 binding. Sterile water was used as positive controls, while wells containing peptide without bacterial suspension were served as negative controls. The active strains were coated on a petri dish, then filter paper with 25 uM Casein 41 were covered on it and incubated overnight. The filter paper with sterile water was added as the negative control. For the microscopic assessment of Casein 24 activity, bacteria sample was treated with SYTO 9 and propidium iodide stains mixture (live/dead^®^ Baclight^™^ bacterial viability kit, Thermofisher, USA) following the manufacturer's instruction. Microscope images were captured by using a fluorescence microscope (Zeiss, Imager.A2, Germany). Bacterium suspension without any disposition were served as negative controls. All experiments were repeated three times with nearly identical results.

### Cell proliferation assays of Casein 89

Human preadipocytes were proliferated in Preadipocyte Medium (PAM, ScienCell Research Laboratories) containing 10% fetal bovine serum (FBS) and 1% penicillin/streptomycin. FITC-labelled Casein 89 synthesised from Science Peptide Biological Technology CO.LTD was added into the medium at 60% confluence, fluorescent was detected after 6 h culture using a fluorescence microscope. The Cell Counting Kit-8 (CCK-8, Dojindo, Tokyo, Japan) experiment was carried out in 96-well plate to determine the proliferation promotion effect of peptide Casein 89. Moreover, a real-time cell proliferation assay was performed using the xCELLigence^®^ impedance-based, label-free, real time cell analysis system (ACEA Biosciences, San Diego CA, USA). Cell indices were plotted against cultured time as growth curves. Cells were plated in 10 % FBS media at the indicated density in 16 microtiter untreated E-plates (ACEA Biosciences, San Diego CA, USA). Peptide Casein 89 treatments were initiated after 4 h incubation. Cell state changes were continuously monitored every hour for a period of 72 h. The peptide solvent phosphate buffer saline was used as a positive control.

### Statistical analysis

Data from the experiments were analyzed by Student's unpaired or paired *t*-tests where appropriate with a significance of *P < 0.05*. The threshold value we used to screen differentially expressed peptides is a fold change ≥ 3.0 or ≤ −3.0 (*P < 0.01*). All data were expressed as the mean ± standard deviation (SD) in this study.

## SUPPLEMENTARY MATERIALS FIGURE AND TABLE





## Abbreviations

CS: cesarean section
LMW: low molecular weight
MWCO: molecular weight cut-off-LC/MS liquid chromatography/mass spectrometry
Casein 24: peptide located at β-Casein 24-38 AA
Casein 89: peptide located at κ-Casein 89-109 AA
MW: molecular weight
PI: isoelectric point
CASA: α-casein
CSN3: κ-casein
PIGR: polymeric immunoglobulin receptor
C4A: complement C4-A
BAL: bile salt-activated lipase
MUC4: mucin-4
ADAM-TS 13: a disintegrin and metalloproteinase with thrombospondin motifs 13
ADFP: perilipin-2
Alpha-2-M: alpha-2-macroglobulin
BSP II: mothers against decapentaplegic homolog 1
TNC: cytotactin
FGB: fibrinopeptide B
FGA: fibrinopeptide A
N-terminal: amino terminal end
C-terminal: carboxyl terminal end
L: leucine
P: proline
MIC: minimal inhibitory concentration
NICU: neonatal intensive care unit
DM1: Type-1 diabetes
DM2: Type-2 diabetes
GDM: gestational diabetes mellitus
SPE: solid phase extraction
FBS: fetal bovine serum
CCK-8: cell counting kit-8
SD: standard deviation
OGTT: oral glucose tolerance test
