# Supplementary material for: Peptidome analysis of human milk from women delivering macrosomic fetuses reveals multiple means of protection for infants
Source: Oncotarget. 2016 Aug 23;7(39):63514–25. doi: 10.18632/oncotarget.11532 (PMC5325381; doi:10.18632/oncotarget.11532)
Supplement: Supplementary file 1 [file oncotarget-07-63514-s001.pdf]

Peptidome analysis of human milk from women delivering macrosomic fetuses reveals multiple means of protection for infants

Supplementary Materials

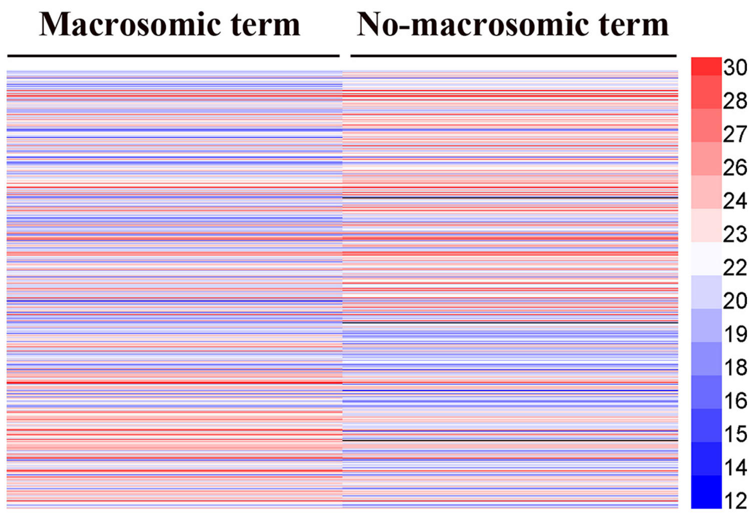

Supplementary Figure S1: Hierarchical clustering shows a remarkable peptide expression profile change between macrosomic group and non- macrosomic group.

Supplementary Table S1: All peptides identified from Mass Spectrometry (MS) analysis. (See Supplementary_Table _S1)
